# Feeding Approach to Optimizing Nutrition in Infants with Congenital Heart Disease

**DOI:** 10.3390/jcdd12020038

**Published:** 2025-01-22

**Authors:** Belinda Chan, Anne Woodbury, Libbi Hazelwood, Yogen Singh

**Affiliations:** 1 Department of Pediatrics, University of Utah, Salt Lake City, UT 84108, USA; 2 Department of Pediatrics, Division of Neonatology, University of California Davis Health, Sacramento, CA 95817, USA; 3 Nutrition Service, University of Utah Hospital, Salt Lake City, UT 84112, USA

**Keywords:** congenital heart disease (CHD), enteral feed (EN), parenteral nutrition (PN), necrotizing enterocolitis (NEC), pulmonary overcirculation, hypoperfusion, hypoxia, malnutrition, feeding strategies, feeding protocol

## Abstract

Congenital heart disease (CHD) affects 1% of live births globally. Infants with CHD often experience growth faltering and malnutrition due to increased metabolic demands, malabsorption, and feeding intolerance, further worsened by surgical interventions and frequent hospitalizations. Malnutrition in this population is linked to higher morbidity, extended hospital stays, and poor neurodevelopmental outcomes. The physiological diversity among CHD types presents significant challenges in developing a universal feeding strategy to optimize nutrition. This narrative review explores the interplay between CHD physiology and nutritional management. CHD types could be categorized into three hemodynamic groups—systemic hypoperfusion, global hypoxia, and pulmonary overcirculation—which help to consider a feeding approach based on such physiology. Nutritional management in these infants could be further tailored based on the disease severity, co-morbidities, and evolving hemodynamic changes. Based on clinical opinions, this review proposes a hemodynamic-focused risk-stratified feeding approach, considering ways that may enhance growth while possibly minimizing complications such as necrotizing enterocolitis (NEC), pulmonary overload, and worsening heart failure. This approach may help individualize nutritional management to address the complex needs of infants with CHD. Further quality improvement studies are needed to assess this approach. Beyond meeting macronutrient needs, micronutrients, including zinc, thiamine, magnesium, vitamin A, and calcium, potentially play a role in cardiovascular health. Given the complexity of nutritional management in these infants, a multidisciplinary team may be needed to optimize care, including cardiologists, neonatologists, pediatricians, dietitians, speech therapists, and pharmacists. With the current knowledge gap and lack of strong evidence, research should focus on nutritional interventions and study their potential impact on infant outcomes with CHDs.

## 1. Introduction

Congenital heart disease (CHD), impacts approximately 1% of live births worldwide. Infants with CHD frequently experience growth faltering and nutritional challenges. These issues stem from increased metabolic demand, feeding intolerance, and malabsorption with further exacerbation from recurrent hospitalizations and surgical procedures [1,2]. Malnourished infants with CHD face higher risks of short- and long-term complications, extended hospital stays, and impaired neurodevelopmental outcomes [2,3].

Standardized feeding protocols have demonstrated benefits, including reduced necrotizing enterocolitis (NEC) risk and improved clinical outcomes [4,5]. Standardization can be achieved through specific guidelines, such as optimizing parenteral nutrition to meet the infant’s metabolic demands [4] or limiting enteral feeding advancements while ensuring the strict use of breastmilk to minimize complications [5]. While neonatal intensive care units (NICUs) are familiar with various feeding protocols, these may be less common in pediatric cardiac ICUs [6]. However, most protocols are not tailored to the different physiological needs of CHD patients. The wide spectrum of CHD types and their diverse clinical trajectories pose significant challenges to developing standardized feeding protocols. CHD can result in complications such as hypoxia, pulmonary overcirculation, or systemic hypoperfusion, each contributing to unique nutritional risks. Consequently, a personalized approach to nutrition is essential to address each patient’s specific needs, mitigate individual risk factors, and promote optimal growth and development. Close collaboration between cardiologists, pediatricians, and dietitians is critical for determining the best medical nutrition therapy course. This review article is focused on the nutritional needs and feeding-related risks in infants with CHD and proposes a hemodynamic-focused, risk-stratified feeding approach that may optimize the growth in infants with CHD.

## 2. Proposed Hemodynamic-Focused, Risk-Stratified Feeding Approach

### 2.1. CHD Categories

Infants with CHD often have increased work of breathing and increased heart rate, leading to increased metabolic demands. In addition, they have varying abnormal blood flow patterns influencing metabolic demand and the hypoperfusion risk associated with enteral nutrition (EN). Cardiovascular effects on end-organ perfusion and oxygenation can change dynamically depending on lesion complexity, CHD severity, and postnatal adaptation. The proposed hemodynamic-focused feeding approach simplifies CHD physiology into three main categories: systemic hypoperfusion, global hypoxia, and pulmonary overcirculation [7,8] (Table 1).

- Systemic hypoperfusion: Reduced blood flow to systemic organs limits oxygen and nutrient delivery. This is often caused by structural left-sided obstructions or imbalances caused by systemic blood flow diverting to pulmonary circulation.
- Global hypoxia: less oxygen is delivered to tissues due to right-sided obstructions, right-to-left shunting, or mixing of oxygenated and deoxygenated blood.
- Pulmonary overcirculation: Excessive pulmonary blood flow caused by left-to-right shunts at the levels of atria, ventricles, or great vessels. This can lead to lung congestion and breathing difficulties.

Subsequently, individual cases are risk stratified based on disease severity, co-morbidities, and evolving hemodynamic changes, as illustrated in the examples and strategies described below.

### 2.2. CHD Severity

The nutritional risk associated with CHD is closely linked to its severity. For instance, a fetal risk stratification pathway for coarctation of the aorta (CoA) has been developed based on prenatal echocardiography findings [9]. This pathway allows early risk assessment, avoids unnecessary interventions, promotes early feeding in low-risk cases, and reduces intestinal strain in high-risk cases [9]. The risk categories estimate the likelihood of a critical CoA requiring immediate surgical correction, as systemic perfusion diminishes with worsening CoA and blood flow restriction. The echocardiography findings critical for evaluating lesion severity and potential for progressive obstruction, particularly in the presence of a large patent ductus arteriosus (PDA), include aortic and valve measurements, biventricular size, retrograde flow, and other associated anomalies [9]. Based on the severity and the index of suspicion for aortic stricture, we propose tailored risk-stratified feeding strategies in infants with confirmed CoA (Table 2).

### 2.3. Dynamic Changes over Time

Hemodynamic effects may evolve into different physiology categories over time or become progressively worse. For instance, infants with atrioventricular canal defects (AVSD) or truncus arteriosus may initially have balanced pulmonary-to-systemic blood flow due to transitional pulmonary hypertension. However, as pulmonary vascular resistance decreases, pulmonary overcirculation can develop, leading to pulmonary hypertension, vascular remodeling, and hypoxia. Similarly, preterm infants with Hypoplastic Left Heart Syndrome (HLHS) who require prolonged prostaglandin E1 (PGE1) to maintain ductal patency are at risk of imbalanced circulation. Initially, these infants are at risk for systemic hypoperfusion, but over time, they may also experience pulmonary overcirculation as they await surgery. After stage 1 palliative HLHS surgery, feeding complications can arise from circulatory adaptations, chylothorax, or vocal cord paralysis. The feeding strategy should be adjusted according to the hemodynamic changes and their clinical effects.

### 2.4. Co-Morbidities

Comorbidities like birth asphyxia, prematurity, small for gestational age (SGA), intrauterine growth restriction (IUGR), chromosomal abnormalities, extracardiac anomalies, infections, delayed enteral nutrition, and prolonged antibiotics can increase the risk of malnutrition and feeding intolerance [7]. Cardiac hypertrophy and arrhythmias may further raise metabolic demands.

## 3. Monitoring

### 3.1. Monitoring Systemic Hypoperfusion and Hypoxia

**Figure 1:**
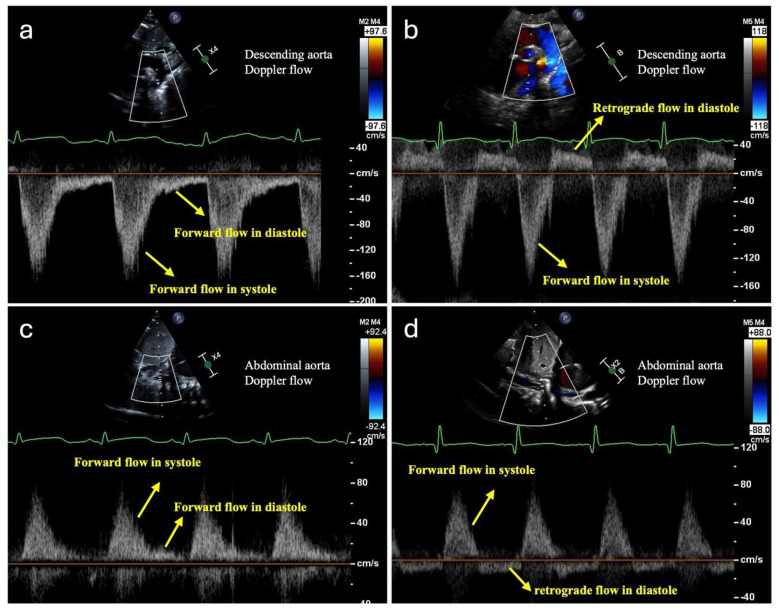
Doppler flow images of descending aorta with the normal forward flow in diastole (**a**) and with the abnormal retrograde flow in diastole (**b**), and of abdominal aorta with the normal forward flow in diastole (**c**) and with the abnormal retrograde flow in diastole (**d**).

**Figure 2:**
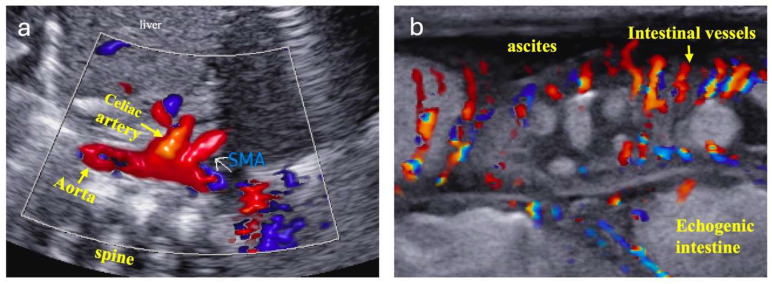
The abdominal ultrasound with color Doppler flow imaging (**a**) highlights the aorta, celiac artery, and superior mesenteric artery (SMA). Doppler assessment (**b**) reveals hyperemia within the small vessels surrounding the intestinal walls, consistent with early-stage necrotizing enterocolitis (NEC). Additionally, ascites and echogenic intestines were observed. Perfusion may eventually cease in the necrotic bowel.

Infants with systemic hypoperfusion and hypoxia physiology are at increased risk of NEC and feeding intolerance, requiring close monitoring. Intestinal perfusion is assessed using physical exams, vital signs, lab tests, and ultrasonography/echocardiography. Signs of intestinal ischemia include abnormal abdominal exams, vital sign changes, and rising lactic acidosis. Echocardiography may show worsening blood flow obstruction, systemic and pulmonary circulation imbalance, and cardiac dysfunction. Doppler ultrasound can detect reduced, absent, or reversed blood flow in the descending aorta, celiac artery, and superior mesenteric artery (SMA), suggesting decreased intestinal perfusion during diastole (Figure 1). Abdominal ultrasound may be more sensitive than X-ray for detecting NEC as it can reveal disrupted bowel structure, absent bowel peristalsis, pneumatosis, complex ascites, and portal venous gas [10]. Doppler flow imaging can demonstrate small vessel perfusion within or around the intestinal wall (Figure 2), showing initially increased intestinal wall blood flow due to inflammation, followed by decreased flow as necrosis develops [10].

Near-infrared spectroscopy (NIRS) measures regional tissue oxygen saturation (rSO_2_) by comparing oxygenated and deoxygenated hemoglobin. Low rSO_2_ indicates insufficient oxygen delivery to meet metabolic demands; a decreasing trend in splanchnic and renal rSO_2_ can signal reduced mesenteric blood flow. A recent meta-analysis found significantly lower rSO_2_ levels in infants with NEC than controls, even before NEC was recognized on clinical exams [11]. NIRS may help predict NEC and guide decisions on initiating or advancing enteral nutrition [12].

### 3.2. Monitoring Pulmonary Overcirculation

**Figure 3:**
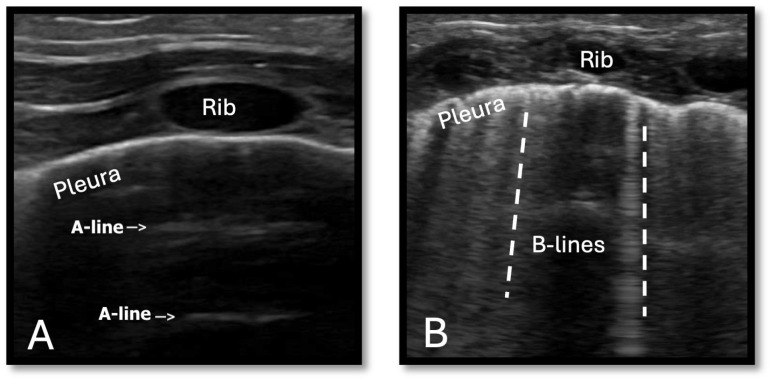
Lung ultrasound images demonstrate (**A**) normal lungs, characterized by echogenic horizontal A-lines (indicated by arrows), and (**B**) lungs with pulmonary edema, identified by multiple echogenic vertical B-lines (indicated by dotted lines).

Pulmonary overcirculation often presents as tachypnea, increased work of breathing, and other respiratory distress signs such as nasal flaring, increased oxygen demand, or respiratory support. Traditional monitoring includes respiratory rate, pulse oximetry, chest X-ray, and blood gasses, while echocardiography provides a detailed pulmonary-to-systemic blood flow ratio. Lung ultrasound (LUS) offers a non-invasive alternative for evaluating pulmonary edema. On LUS, normal air-filled lung parenchyma appears as horizontal echogenic lines (A-lines) from the pleura’s artifact reflection. B-lines appear as echogenic vertical lines resulting from reverberation artifacts. These lines indicate the presence of interstitial and alveolar fluid, with an increased number of B-lines reflecting increased pulmonary congestion [13] (Figure 3). A prospective study by Kaya et al. showed that LUS B-lines findings correlate with pulmonary edema in infants with CHD, as demonstrated by chest x-ray findings and increased pulmonary blood flow on Doppler perioperatively [14].

## 4. Enteral Nutrition Feeding Strategies

### 4.1. Overview

**Figure 4:**
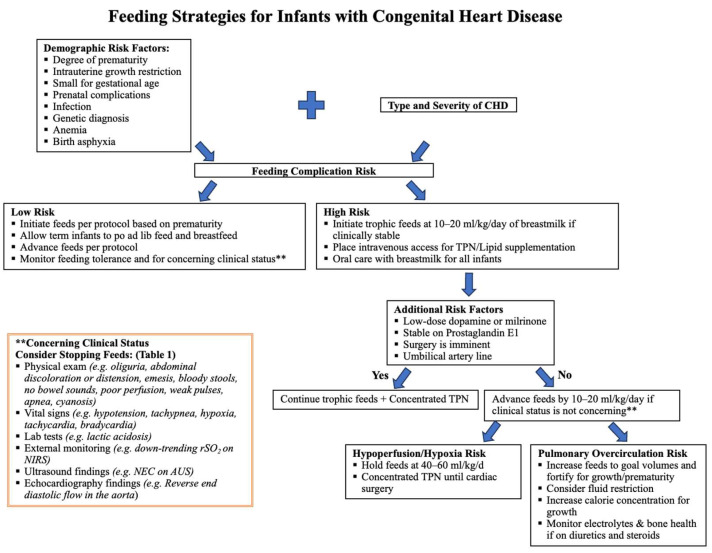
The proposed hemodynamic-focused, risk-stratified feeding flow chart is based on comorbidities, CHD type, and CHD severity.

Over the past decades, cardiovascular surgery success rates for infants have improved, allowing for a shift of focus from mere survival to enhanced overall outcomes [15]. Malnutrition in pediatric patients before cardiac surgery has been linked to increased morbidity, mortality, infections, and prolonged hospital stays [1]. For infants with CHD, it is essential to maintain a careful balance between maximizing nutritional provision and preventing NEC and fluid overload. We categorize CHD based on hemodynamic physiology (Table 1) and present an example of risk-stratified management using the severity of CoA as a model (Table 2). Combining these principles, we propose a hemodynamic-focused, risk-stratified feeding flowchart tailored for infants with CHD (Figure 4).

### 4.2. Trophic and Limited Feeds—For Infants at Risk for Hypoperfusion and Hypoxia

Holding at trophic feeding volumes is a feeding strategy for infants at risk of cardiac NEC due to hypoperfusion and hypoxia. Cardiac NEC is a severe feeding complication affecting infants with CHD. Cardiac NEC differs from preterm NEC in that it may occur in term infants, typically affects the colon instead of the small intestine, and can be more severe [16]. The modifiable risk factors for cardiac NEC include feeding volume, advancement rate, and type of feed. The non-modifiable factors are prematurity, genetic syndromes, intrauterine growth restriction (IUGR), and being small for gestational age (SGA) [16,17,18]. Infants with CHD frequently experience chronic inflammation and hypoxia, with recurrent hypoxic events fostering anaerobic bacterial overgrowth and increasing the risk of mucosal damage and pneumatosis intestinalis in the gastrointestinal tract [16,17,18]. These factors, combined with low cardiac output and specific cardiac lesions, further impair intestinal perfusion [16,17,18].

While trophic feeds may elevate intestinal metabolic demand, research suggests that early enteral stimulation can reduce the risk of NEC [18,19]. Trophic feeds support intestinal immunity, suppress pathogenic intestinal flora, and prevent intestinal atrophy [18,19]. Evidence indicates that infants may safely receive trophic feeds even while on PGE1, low-dose inotropes, or with an umbilical arterial catheter (UAC) in place [19,20,21].

PGE1 may alter splanchnic blood flow by redirecting systemic circulation to the pulmonary system, potentially leading to intestinal hypoperfusion. A recent study by Mankouski et al. showed that preterm infants with CHD on PGE1 exhibit lower splanchnic regional oxygen saturation (rSO_2_), suggesting impaired intestinal perfusion [22]. Thus, while feeding may be safe under stable conditions, close monitoring for signs of intestinal ischemia is essential. Similarly, some centers administer trophic feeds to infants on stable, low-dose cardioactive medications, such as dopamine, dobutamine, or milrinone. However, feeds should be paused if hemodynamic instability occurs or inotropic support is escalated. Finally, in a prospective study featuring 19 infants with a mean gestation of 27 weeks, Hevrank et al. reported no major changes in mesenteric blood flow when trophic feedings were administered with a UAC present and after UAC removal [21]. Therefore, the European Society of Paediatric and Neonatal Intensive Care (ESPNIC) supports trophic feeding with UAC for clinically stable infants [23].

For stable, low-risk infants with CHD, gradual feeding advancement beyond trophic feeding is possible with close monitoring for signs of intolerance. Ideally, allowing term infants to feed orally ad-lib based on cueing. It may also be beneficial to gradually increase enteral feeds in high-risk infants up to 40–60 mL/kg/day, as recommended by Kataria-Hale et al. [6]. In their single-center, quasi-experimental study, implementing a standardized presurgical feeding protocol led to higher enteral feeding volumes before surgical repair without an increase in adverse outcomes. Additionally, after protocol initiation, more infants received human milk rather than formula [6]. However, Cognata et al. showed that a larger enteral feeding volume of >100 mL/kg/day may be associated with NEC [24]. Maintaining lower feeding volumes when at risk for NEC due to hypoperfusion helps reduce intestinal strain before corrective cardiac surgery. After surgery, feedings can be advanced to full volume and fortified to optimize growth, as the risk of NEC is lower with improved perfusion and flow dynamics.

Many centers give bolus feeds every 3 h to infants who cannot feed orally because this supports natural digestive hormone release [6]. Mankouski et al. showed no significant change in the pre- or post-prandial rSO_2_ in preterm infants on PGE1 with bolus feeding [22], suggesting bolus feeding can be safe. Some centers opt for continuous feeds to reduce intermittent intestinal metabolic demand; however, this may increase nutrient losses due to fat and protein adhering to the tube. Mills et al. reviewed multiple studies and concluded that transpyloric feeding is feasible and delivers more enteral calories; however, it does not provide additional protection against aspiration or reduce other complications [19].

Breastmilk is the best form of nutrition for infants with CHD [19], as it contains antimicrobial antibodies, such as IgA and IgG. It has fewer pro-inflammatory agents than bovine milk and includes interleukins and prostaglandins that protect the intestinal mucosa [6]. Breastmilk also provides natural digestive enzymes, prebiotics, and probiotics, which promote the growth of healthy microvilli. Breastmilk is associated with a lower risk of NEC than cow milk formula in infants with CHD [24]. When breastmilk or pasteurized donor milk is unavailable, some centers use semi-elemental or elemental formulas as they may be absorbed more efficiently with better tolerance than standard term formulas [4,5,23]. However, data supporting the use of these formulas for CHD are lacking.

### 4.3. Feeding Strategies for Infants with Pulmonary Overcirculation

Infants with CHD and pulmonary overcirculation may experience feeding fatigue and tachypnea, leading to increased caloric expenditure and failure to thrive. Fluid restriction can help prevent cardiorespiratory overload, but higher caloric concentrations, up to 27–30 kcal/oz, are often required for growth. Again, vigilant monitoring of feeding fortification tolerance vs. NEC risk is prudent. Diuretics may prevent fluid overload but may also cause electrolyte imbalances, dehydration, osteopenia, and bone mineral disease (MBD) [25], necessitating close blood test monitoring.

## 5. Parenteral Nutrition (PN) Strategies

**Figure 5:**
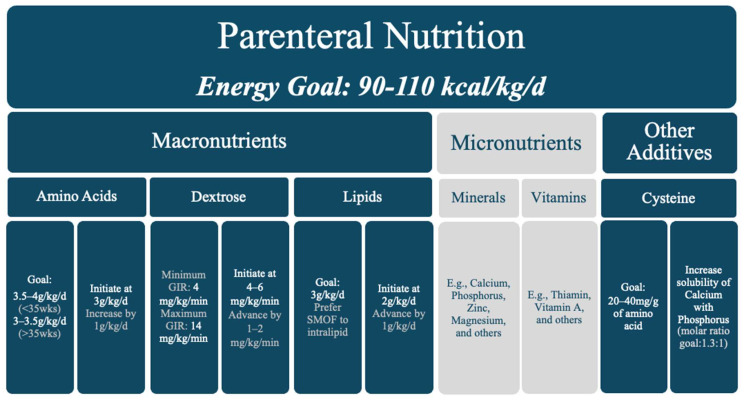
Parental nutrition composition for infants with CHD.

Infants with CHD typically need 90–120 kcal/kg/day and 3.5–4 g/kg/d of protein, with many having even higher needs [6,19]. When infants with CHD are limited to trophic or low-volume enteral feeds, PN provides essential nutrients for growth and better perioperative outcomes. PN can be a lifesaving intervention to prevent excessive catabolism, hypoglycemia, and micronutrient deficits [26]. Many studies have published recommended PN calorie, protein, carbohydrate, and fat composition, as summarized in Figure 5 [6,25]. PN should be optimized nutritionally to ensure the least hepatic burden possible.

PN risks include liver disease, electrolyte and nutrient imbalance, central line infection, and digestive tract cell atrophy. To reduce liver injury, use PN products with sodium and phosphate additives containing the lowest aluminum levels, varying from 180 to 31,000 mcg/dL across brands [27]. SMOFlipid^©^ is hepatoprotective by reducing the rate of PN-related cholestasis and lowering direct bilirubin levels, while fish oil-based lipids can be therapeutic in addressing hepatic dysfunction [28]. Targeting adequate calorie and protein intake, adjusting osmolarity, balancing macronutrients, optimizing electrolytes, and monitoring growth are essential while awaiting CHD surgery on PN [23]. Trophic feeding helps prevent intestinal disuse and atrophy. Maintaining strict vigilance with central line sterility and ensuring prompt removal of central lines are crucial to minimizing the risk of infection.

## 6. The Overlooked Importance of Micronutrients for Cardiovascular Health

Most published EN and PN protocols emphasize macronutrients, however micronutrients may also play a vital role in supporting cardiovascular health in infants with CHD. Adequate intake of vitamins and minerals may enhance cardiac function as they scavenge free oxygen radicals, and may boost myocardial energy production. The limited evidence that suggests the effect of these micronutrients on cardiovascular function is discussed here [29,30,31,32,33,34,35,36,37,38,39,40,41,42,43,44,45,46,47,48,49,50].

### 6.1. Zinc

Zinc mitigates oxidative stress by inducing metallothionein synthesis, sequestering reactive oxygen species (ROS), and acting as a cofactor for redox enzymes [29,30,31,32,33,34,35]. It also inhibits NADPH oxidase and regulates proteins involved in inflammation, apoptosis, and nitric oxide signaling [29,30,31,32,33,34,35]. In the context of pediatric congenital heart disease (CHD), studies have demonstrated significantly lower serum zinc levels in affected infants compared to healthy controls, suggesting a potential link between zinc deficiency and the increased risk of CHD [30]. Zinc deficiency may impair tissue repair and immune function by lowering TGF-β1 expression, complicating CHD post-surgery recovery [29,30,31,32,33,34,35]. Infants with CHD are at risk of deficiency because of diuretic therapy, such as thiazides and ACE inhibitors, which can cause zincuria [32,33]. Prolonged PN dependence and declining zinc levels in breast milk further challenge maintaining adequate zinc levels [34]. The signs of zinc deficiency include delayed wound healing, seborrheic dermatitis, and diarrhea [29].

### 6.2. Thiamine (Vitamin B1)

Thiamine facilitates energy production and proper heart function by supplying adequate ATP for cardiac contractions and energy transport [36,37,38]. Deficiency causes mitochondrial dysfunction, cardiac hypertrophy, heart failure, and lactic acidosis, often mimicking sepsis and potentially leading to fatal outcomes if undiagnosed [36,37,38]. A recent case report described a 2-month-old infant diagnosed with infantile beriberi, presenting with right heart failure and lactic acidosis caused by thiamine deficiency [37]. In PN-dependent infants, monitoring and replenishing thiamine may be important, particularly in maternal deficiency or malnutrition that compromises uteroplacental transfer [36,37,38]. Signs of deficiency include bilateral leg edema and peripheral neuropathy muscle weakness [36,37,38].

### 6.3. Vitamin A

Vitamin A, one of the most common micronutrient deficiencies, may affect fetal heart development [39,40]. Maternal Vitamin A deficiency has been linked to fetal congenital heart abnormalities [40]. Retinoic acid supports the formation of heart structures such as septa, atrioventricular cushions, epicardium, ductus arteriosus, and ventricular muscle and has potential therapeutic applications in myocardial regeneration [40]. It also affects myocardial function and cardiovascular disease through signaling pathways, and it offers antioxidant protection by reducing oxidative stress-induced apoptosis during myocardial ischemia and reperfusion injury [39,40,41]. Vitamin A deficiency can present as night blindness, white/gray spots in the eyes, corneal softening, and diarrhea [42].

### 6.4. Magnesium

Magnesium supports cardiovascular function and cardiac rhythm regulation [43,44,45,46,47]. It acts as a coenzyme, antioxidant, and calcium antagonist, particularly in conditions like myocardial ischemia [43,44,45,46,47]. As a calcium antagonist, magnesium protects against arrhythmias and ischemia by preventing calcium overload, reducing tachycardia, and helping muscle excitation-contraction coupling of the heart [45]. Magnesium also modulates ATP-dependent reactions and energy generation for cardiovascular function [43,44,45,46,47]. Diuretic therapy may deplete electrolytes, including magnesium [43,44,45,46,47]. If hypokalemia, hyponatremia, and hypophosphatemia occur, obtaining a serum magnesium level and addressing repletion is encouraged before increasing other electrolytes [43,44,45,46,47]. Magnesium deficiency can also present as hypertension, loss of appetite, jitteriness, feeding intolerance, and seizures [47].

### 6.5. Calcium

Calcium plays a role in myocardial contraction, vascular tone regulation, and nerve signal transmission [48,49]. Deficiency can lead to cardiovascular symptoms such as cardiac arrhythmias, hypotension, and, in severe cases, heart failure [25,48,49]. Other symptoms include muscle cramps, tetany, numbness, muscle weakness, seizure, osteopenia, and more. In addition to diuretics and steroids, prolonged PN can elevate the risk of metabolic bone disease (MBD), partly due to aluminum contamination [27]. This risk is further compounded when provisions of calcium and phosphorus are insufficient to support mineral accretion [25]. Adding cysteine to PN can help maximize calcium and phosphorus delivery by increasing the solubility of the solution [50].

## 7. Nutrition-Focused Physical Exam and Growth Assessment

A practical feeding strategy should support adequate growth, assessed by serial weight, length, and head circumference measurements. Along with meeting calorie needs for weight gain, protein intake must be sufficient to support linear growth, muscle development, and organogenesis. Nutrition-focused physical exams (NFPE) offer a more comprehensive growth assessment, evaluating areas for muscle and fat loss, as shown in Table 3 [51].

## 8. Standardized Feeding Protocols

After understanding the nutritional challenges for infants with CHD, each institution should consider developing standardized feeding protocols based on hemodynamic risk factors. Evidence shows that feeding protocols can reduce NEC risk, lower mortality, and morbidity, decrease IV access days, prevent line infections, shorten PN use, and reduce the length of stay [6]. Although larger studies on optimal feeding strategies are lacking, individual centers’ presurgical CHD feeding protocols may serve as valuable references [6,17,52,53,54,55]. For example, in a single-center cohort study, O’Neal Maynord et al. demonstrated that the implementation of a preoperative and postoperative feeding program for infants with CHD led to significant improvements in weight-for-age z-scores at hospital discharge, increased preoperative enteral feeding, and reduced gastrostomy tube placement. Their multi-faceted preoperative nutrition approach, which included utilizing donor human milk when parental milk was unavailable and an emphasis on oral feeding assessment before surgical repair, resulted in increased preoperative enteral feeding volumes and improved oral feeding post-surgery. Importantly, these interventions did not increase necrotizing enterocolitis (NEC), length of stay (LOS), or mortality [52]. Similarly, Furlong-Dillard et al. found earlier enteral feeding perioperatively and shorter PN use postoperative after feeding protocol implementation for neonates who underwent biventricular cardiac surgery [53].

We believe feeding plans could be based on the type and severity of CHD with input from multiple disciplines. Compared to other studies, the advantage of our proposed hemodynamic-focused, risk-stratified approach is that the feeding strategy can be adjusted as the infants’ hemodynamic physiology changes based on echocardiography findings. Further quality improvement studies are needed to assess this approach. Regardless of the feeding protocol used, consistent adherence and minimizing variation may be crucial for improving outcomes and reducing the risk of NEC [6,54,55].

## 9. Conclusions

Optimizing nutrition is pivotal for improving both short- and long-term outcomes in infants with CHD. This review highlights the importance of tailoring feeding strategies based on cardiac diagnoses and hemodynamic findings, including risks of systemic hypoperfusion, global hypoxia, and pulmonary overcirculation. Nutrition-focused interventions, including micronutrient supplementation, may be essential for supporting cardiovascular health and overall growth. There is still a major knowledge gap and limited evidence in the nutritional management of infants with CHD. Research should prioritize refining individualized feeding protocols, implementing quality improvement projects, following long-term outcomes, and investigating the role of micronutrients. A multidisciplinary collaborative approach is critical to advancing care and promoting optimal growth in this vulnerable population.

## Figures and Tables

**Table 1:** A proposed hemodynamic-focused, risk-stratified feeding approach to optimize nutrition for infants with congenital heart disease.

	Systemic Hypoperfusion	Global Hypoxia	Pulmonary Overcirculation
Causes	Left-sided obstructions Imbalance of systemic perfusion from pulmonary steal (Qp > Qs)	Right-sided obstructions Intracardiac mixing between oxygenated and deoxygenated blood Right-to-left shunting	Increased left-to-right shunt at the levels of atria, ventricles, or great vessels (Qp > Qs)
Examples of Cardiac lesions	*Left Obstructions:* Hypoplastic left heart, Coarctation of aorta *Imbalance:* Double outlet left ventricle	*Right Obstructions:* Pulmonary atresia, Tetralogy of Fallot *Central mixing:* Transposition of great vessels, Total anomalies pulmonary venous connection, truncus arteriosus *Right-to-left shunt:* Ebstein Anomaly	*Left-to-right shunt:* Atrioventricular canal defects, Aortopulmonary window, Ventricular septal defect, Patent ductus arteriosus, atrial septal defects
Hemodynamic and end-organ effects	Decreased systemic blood flow to vital organs, including intestines, kidney, and brain.	Decreased global oxygen delivery to vital organs	Increased pulmonary blood flow causing respiratory compromise Increased energy expenditure Chronic pulmonary overcirculation may lead to pulmonary hypertension
Signs and symptoms	Hypotension Weak femoral pulses Delayed cap refill Differential upper and lower blood pressure Feeding intolerance (e.g., abdominal distension, bloody stool, bilious emesis) Metabolic acidosis Oliguria	Cyanosis Metabolic acidosis Hypoxia	Tachypnea Respiratory distress Hepatomegaly Hypoxia
ECHO parameters	Reduced or reverse end diastolic flow at descending Aorta, Celiac and Superior Mesenteric arteries Decreased ventricular function and contractility Decreased LV cardiac output	Right-to-left Doppler flow at atria, ventricle, or great vessel level Decreased RV cardiac output	Increased pulmonary flow Left atrium and ventricle dilation Left-to-right Doppler flow at atrial, ventricular, or great vessels level
External monitoring	Near-infrared spectroscopy Blood gas Serum lactate Vital signs Abdominal X-ray Abdominal ultrasound for intestinal pathology Aortic Doppler flow Urine output	Near-infrared spectroscopy Pulse Oximetry Blood gas Fraction Oxygen requirement	Chest X-ray Lung Ultrasound Blood gas Intake and output fluid balance Daily weight Respiratory support
Feeding considerations	Lower metabolic demand Limit hemodynamic stress on intestine Parenteral nutrition for supplementation, while monitoring for associated complications and cholestasis Breastmilk or elemental formula	Lower metabolic demand Limit hemodynamic stress on intestine Parenteral nutrition for supplementation, while monitoring for associated complications and cholestasis Breastmilk or elemental formula	Same feeding considerations for hypoperfusion/hypoxia due to pulmonary steal Fluid restriction Maximize caloric density Monitor electrolyte imbalance and for osteopenia while on diuretics

**Table 2:** Coarctation risk stratification and management.

	Low Suspicion	Moderate Suspicion	High Suspicion
Prenatal Echocardiography findings	Transverse arch z-score <−2 and >−2.5 Aortic isthmus z-score <−2 and >−2.5	Transverse arch z-score <−2.5 and >−3 Aortic isthmus z-score <−2.5 and >−3 Isthmus: ductus ratio > 0.7 Pulmonary artery to aorta diameter ratio >2	Transverse arch z-score <−3 Aortic isthmus z-score <−3 End-on-side insertion of isthmus Posterior coarctation ledge Left to right or bidirectional Patent foramen ovale flow
Care plan and feeding strategy	Routine newborn care: Echocardiography before hospital discharge Allow breastfeeding or oral feeding on demand.	Cautious approach until CoA is ruled out: Echocardiography within 12 h after birth No routine Prostaglandin E1 at birth and monitor serum lactate and femoral pulses closely Place peripheral intravenous access Begin D10W at 60–80 mL/kg/day Allow oral feeding and breastfeeding per demand and gestational age, slow advance feed as tolerates Withheld gavage feeding until CoA is either ruled out or found to be low risk based on Echocardiography. Monitor vital signs and perform a pulse check every 3–4 h to detect signs of CoA *	Immediate CoA management: Urgent Echocardiography soon after birth Start Prostaglandin E1 Place central vascular access (e.g., UAC/UVC) Begin D10W at 60–80 mL/kg/day

* Clinical concerns for CoA: systolic upper and lower extremity blood pressure gradient > 20 mmHg, weak femoral pulses, decreased urine output, unexplained tachycardia, and poor perfusion. Abbreviation: CoA Coarctation; D10W 10% Dextrose Intravenous fluid; kg kilogram; mL milliliter; mmHg millimeters of mercury; UAC umbilical arterial catheter; UVC umbilical venous catheter.

**Table 3:** Signs of malnutrition in nutrition-focused physical exams.

Signs of Malnutrition in Nutrition-Focused Physical Exams (NFPE) [51]	
General	Down trending z-score of weight, length, and head circumference Hair loss Skin depigmentation Delayed tooth eruption
Face	Muscle wasting of the temporal region Fat wasting of the orbital or buccal region
Upper Body	Muscle wasting of the triceps, clavicle, or acromion bone region
Upper Back	Muscle wasting of the scapular region
Midaxillary Line	Fat wasting of the thoracic or lumbar region
Lower Body	Muscle wasting of the quadricep, patellar, or gastrocnemius regions

## Data Availability

Not applicable.

## Abbreviations

Arterial oxygen saturation (SaO_2_), Aortic Insufficiency (AI), Aortic Stenosis (AS), Atrial Septal Defect (ASD), Atrioventricular Canal (AV canal), Congenital heart disease (CHD), Birthweight (BW), Coarctation of Aorta (CoA), 10% Dextrose water (D10W), Double outlet right ventricle (DORV), Echocardiography (ECHO), Enteral feeding (EN), Gestational age (GA), Hypoplastic Left Heart syndrome (HLHs), Intrauterine growth restriction (IUGR), IV intravenous (IV), intravenous fluid (IVF), kilogram (kg), Lung ultrasound (LUS), milliliter (ml), millimeter of mercury (mmHg), Near-infrared spectroscopy (NIRS), Necrotizing Enterocolitis (NEC), Nil by mouth (NPO), Nutrition-Focused Physical Exams (NFPE), Patent Ductal arteriosus (PDA), Partial pressure of oxygen (PaO_2_), Partial pressure of carbon dioxide (PCO_2_), Peripheral intravenous access (PIV), Prostaglandin E1 (PGE1), Pulmonary Atresia (PA), Pulmonary Hypertension (PH), Pulmonary Stenosis (PS), pulmonary–systemic flow ratio (Qp > Qs), regional tissue oxygen saturation (rSO₂), Small for gestational age (SGA), Transposition of Great Vessel (TGA), Tetralogy of Fallot (TOF), Total Parenteral Nutrition (TPN), Truncus Arteriosus (TA), Ventricular Septal Defect (VSD), Umbilical Arterial Catheter (UAC), Umbilical Venous Catheter (UAC).
